# PEComa in a Young Patient with Known Li-Fraumeni Syndrome

**DOI:** 10.1155/2015/906981

**Published:** 2015-03-04

**Authors:** Kyriakos Neofytou, Simone Famularo, Aamir Z. Khan

**Affiliations:** Upper GI/HPB Unit, Department of Academic Surgery, Royal Marsden Hospital, Fulham Road, London SW3 6JJ, UK

## Abstract

Perivascular epithelioid cells neoplasms (PEComas) constitute a family of rare tumours which have been reported virtually in all anatomic sites. The histological clarification of the malignant potential of these tumours is still problematic despite the proposed risk stratification systems. Li-Fraumeni syndrome (LFS) is caused by a germline mutation in the TP53 tumour suppressor gene. It is a rare but well-characterized cancer predisposition syndrome leading to the development of a variety of different tumour types. To the best of our knowledge, an association between this syndrome and PEComas has not been previously documented. A 24-year-old lady with known LFS presented with two uncertain-in-nature lesions, one within the right part of the liver and one within the upper pole of the right kidney. The patient underwent an uncomplicated open simultaneous right partial nephrectomy and resection of segment 7 of the liver. The morphological and immunohistochemical features of both lesions were of epithelioid angiomyolipoma (PEComa). Although the obvious scenario was that the liver lesion was a metastasis from the renal lesion, the assessment of their malignant potential according to the existing risk stratification systems was rather in favour of two synchronous primary PEComas, pointing out that the histological assessment of malignant potential of PEComas is still problematic.

## 1. Introduction

Perivascular epithelioid cells neoplasms (PEComas) were first described by Bonetti et al. in 1992. In 2002 PEComas were recognized as an independent entity in accordance with the World Health Organization [1, 2]. PEComas are a family of tumours constituted by angiomyolipoma of the kidney (AML), clear cell sugar tumor of the lung (CCST), lymphangioleiomyomatosis of the lung (LAM), and perivascular epithelioid cell tumor not otherwise specified (PEComa-NOS). PEComa-NOS are very rare tumours in other anatomical sites and have been described in virtually every anatomic site including colon, pancreas, retroperitoneum, heart, adrenal gland, breast, eye, biliary tract, bone, urinary bladder, skull base, cervix, skin, nasopharynx, and liver [2, 3].

The diagnosis of these rare tumours predominately depends on immunohistochemistry and the characteristic coexpression of melanocytic (HMB45 and/or melan A) and smooth muscle markers (actin and/or desmin) [4–6]. The histopathologic clarification of the malignant potential of these tumours is still problematic despite the proposed risk stratification systems [4–6]. The only potentially curative option for these patients is surgical resection, while the results of transitional and emerging systemic therapies are still very poor [6].

Li-Fraumeni syndrome (LFS) is rare but very well-characterized cancer predisposition syndrome [7]. The underlying cause of LFS is a germline mutation in the TP53 tumour suppressor gene [8]. Carriers of such mutations have, on average, 50% chance to develop cancer before the age of 40 years, compared with 1% in the general population, and 90% of the carriers are diagnosed with cancer by the age of 60 years [9]. 15% of these patients develop a second, 4% develop a third, and 2% develop a fourth cancer [10].

We report a case of a young patient with known LFS, who developed PEComa. To the best of our knowledge, an association between PEComa and Li-Fraumeni syndrome has not been reported before.

## 2. Case Report

A 24-year-old lady presented with mild pain in the right lumbar region. The physical examination was unremarkable. Standard laboratory test results, including urinalysis and urine culture, were within normal range. Her past medical history included a rhabdomyosarcoma of the right buttock surgically treated at the age of six months and a germiline pathogenic p53 mutation (Li-Fraumeni syndrome). She did not take any regular medication and she was in good general health. An abdominal ultrasound scan revealed two uncertain-in-nature lesions, one within the right part of liver and one within the upper pole of the right kidney.

**Figure 1:**
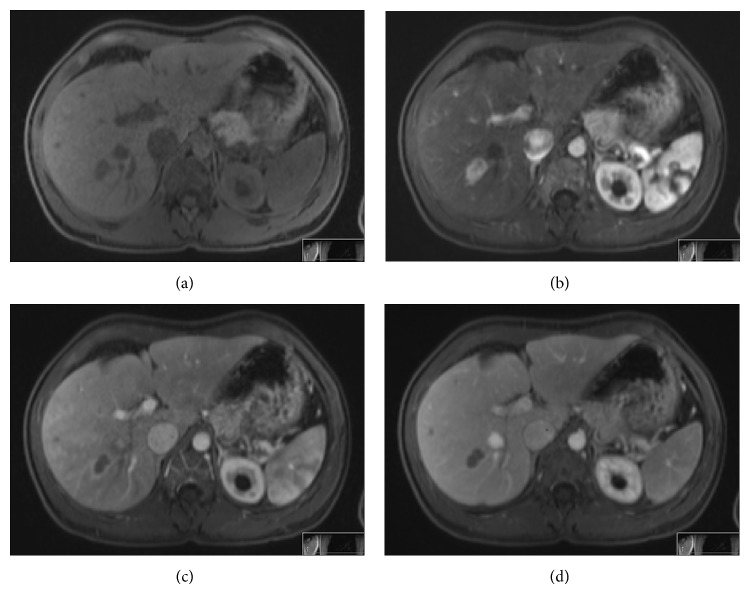
MRI axial T1-weighted images of the liver lesion before and after intravenous contrast medium administration. (a) Preintravenous contrast medium administration; focal lesion in segment VII of the liver and (b) arterial phase; early enhancement following contrast administration, and (c, d) venous and equilibrium phase, respectively, early washout.

**Figure 2:**
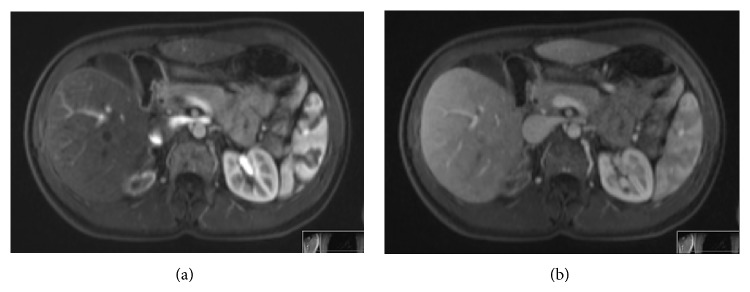
MRI axial T1-weighted images of the right kidney lesion after intravenous contrast medium administration. (a) Arterial phase, peripheral enhancement following contrast administration, and (b) venous phase.

A subsequent MRI scan of liver and kidneys confirmed the presence of the two lesions that were shown at ultrasound scan. The lesion within segment 7 of the liver was 1,8 × 1,3 cm in diameter. It was solid and showed restricted diffusion. It was enhanced early and fairly intensely following contrast administration and also exhibited early washout (Figure 1). The lesion within the upper pole of the right kidney showed peripheral enhancement following contrast administration and demonstrated impeded diffusion at its peripheral aspect (Figure 2).

The MRI features of the two lesions were not pathognomonic for a specific pathologic entity. Following discussion within the liver and urology multidisciplinary team meetings, a recommendation for simultaneous right partial nephrectomy and partial hepatectomy was made (due to the patient's Li-Fraumeni syndrome and the associated high risk of malignancies development).

The patient underwent an uncomplicated open simultaneous right partial nephrectomy and resection of segment 7 of the liver and she was discharged 5 days later.

The morphological and immunohistochemical features of both lesions were of epithelioid angiomyolipoma (PEComa).

Histopathological examination revealed that both lesions were confined to the liver and kidney, respectively, with no evidence of invasion into surrounding tissues. The border between the tumours and the surrounding liver and renal parenchyma, respectively, was well defined. The size of the kidney and liver lesions according to histopathological examination was 30 mm and 10 mm, respectively. There was no lymph node or lymphovascular invasion and both lesions were completely excised.

Both lesions were composed of an admixture of spindle and epithelioid cells, with a predominance of spindle shaped tumour cells (>70% of the tumour cells). Within both lesions there were scattered mature adipocytes and also scattered pleomorphic cells with anaplastic and multinucleated nuclei. Definite tumour necrosis could not be identified within either of the lesions.

Mitotic figures were easily seen only within kidney lesion, amounting to 10 per 10 hpfs with scattered atypical forms being present, while within liver lesion mitotic figures were not identified.

Immunohistochemistry of both lesions shows the tumour cells to strongly express melan A, HMB45, smooth muscle actin (SMA), and caldesmon, with focal S100 expression. There was no expression of cytokeratin-AE1/AE3, cytokeratin-CAM5.2, CD10, CD117, or DOG1. Additionally there was no expression of EMA, PAX8, RCCAg, myogenin, and MyoD1 within the kidney lesion, and there was no expression of CK5/6, Hepar1, AFP, and CD34 within the liver lesion. The proliferation marker Ki67 was expressed in approximately 10 to 20% of the tumour cells within both lesions. The only difference between the two lesions regarding immunohistochemistry was the absence of desmin expression within the liver lesion while there was focal desmin expression within the kidney lesion.

The patient did not receive any adjuvant systemic treatment and, at the last follow-up, 1 year from surgery, she remains disease-free.

## 3. Discussion

Li-Fraumeni syndrome is a cancer predisposition syndrome caused by a germline mutation in the TP53 tumour suppressor gene [7, 8]. While most of the carriers of cancer predisposition syndromes are at increased risk of site-specific cancers, carriers of LFS have been reported to develop a variety of different tumour types such as sarcomas, breast cancers, brain tumors, adrenocortical carcinomas, kidney and liver tumors, leukemias, melanoma, and lung, gastric, and pancreatic cancers [7–10]. An association between this syndrome and PEComas has not been previously documented.

PEComa is a family of rare tumours consisting of perivascular epithelioid cells (PECs) that coexpress melanocytic (HMB45 and/or melan A) and smooth muscle markers (actin and/or desmin) [4–6]. The cell of origin of these tumours remains unknown; that is, normally, no perivascular epithelioid cells exist. Possible progenitors of PECs considered undifferentiated cells of neural crest, differentiated smooth muscle cells, and pericytes [11].

PEComas have been reported in almost every anatomic site and at virtually all ages, with a median age of presentation of 43 years. They present a strong predominance in females, with a female : male ratio of 4 : 1 [4–6]. PEComas exhibit a wide spectrum of clinical and radiographic findings depending on the tumour site. Rarely, these findings can lead to the definitive diagnosis which usually arises after the removal of tumours [12].

The only potentially curative treatment for primary PEComas and for local recurrences and metastasis is surgical resection [6, 13]. Despite the encouraging results of small clinical trials on the effectiveness of oral administration of mTOR inhibitors in patients with metastatic PEComas, in general, PEComas are chemotherapy- and radiotherapy-insensitive tumours [6, 13, 14].

The establishment of malignant potential of PEComas is challenging. Although the vast majority of PEComas show a benign course, some are aggressive with locally destructive recurrences and distant metastasis [4–6].

Until today, none of the three proposed risk stratification systems is worldwide accepted as all of them developed by the retrospective analysis of histopathologic features of a relative small number of cases [4–6]. In 2005 Folpe et al. proposed the first of these stratification systems by analyzing 26 PEComas of soft tissue and the gynecologic tract [4]. This analysis concluded to the stratification of PEComas in three categories: benign, uncertain malignant potential, and malignant, according to the tumour size (cut-off value 5 cm), the mitotic rate (cut-off value > 1/50 HPF), the presence of high nuclear grade and cellularity, infiltrative growth pattern, vascular invasion, and tumour necrosis. Some years later, in 2012, Bleeker et al. evaluated Folpe's criteria in a cohort of 236 cases of PEComa-NOS which were extracted by reported cases until then [6]. Cases of AML, LAM, and CCST were excluded from that analysis. The conclusion of this study was that only tumour size (cut-off value 5 cm) and mitotic rate (cut-off value > 1/50 HPF) were associated with recurrence following surgical resection [6]. In 2010, Brimo et al. proposed a different stratification system following analysis of 40 cases of renal epithelioid AML with atypia resected in three institutions [5]. Based on their findings, they developed a predictive model of 4 atypical features that included ≥70% atypical epithelioid cells, ≥2 mitotic figures per 10 hpf, atypical mitotic figures, and necrosis, concluding that the presence of 3 or all of these features was highly predictive of malignant behaviour. The accuracy of this model to predict the malignant behaviour of a tumour was only 78% [5].

In our case the presence of the liver epithelioid AML proposed that this lesion was a metastasis from the renal lesion, which definitely makes the renal lesion malignant. However, it was difficult to confirm or refute this possibility based on the histopathological findings, with the alternative hypothesis being that they were synchronous tumours.

Assessment of the renal lesion malignant potential was tried despite the difficulties arising from the fact that there are competing grading systems. According to the grading system specifically developed for renal epithelioid AMLs [5], this tumour scores 2 out of four (mitotic count >2 per 10 hpfs, presence of atypical mitoses, but an absence of epithelioid morphology in >70% of the tumour cells or definite tumour necrosis) with scores of 3 or above said to be highly predictive of malignant behavior [5]. However, on this staging system, some 22% of the malignant examples did not score three or greater so that a score of 2 cannot definitely be determined as benign. Such an intermediate score is not further interpreted in this grading system. In the alternative general PEComa grading system this tumour would score 2 (high mitotic count and marked pleomorphism) which would put it unequivocally within the malignant category [4, 6]. The applicability of this late grading system to the kidney is not yet agreed on.

Liver primary AMLs are so rare that specific malignant criteria are not established. Within the Brimo et al. grading system, liver lesion had no features indicating aggressive/malignant potential, but the lesion would be termed of uncertain malignant potential (due to nuclear pleomorphism/multinucleated giant cells) in the more general PEComa grading system [4–6].

All the above clearly demonstrate the weakness of the existing risk stratification systems for PEComas. Even in a case such as ours, where the presence of a second metastatic tumour definitely makes the renal lesion malignant, this cannot be deduced safely based only on the histological features of the renal lesion.

## 4. Conclusions

PEComas are rare tumours which develop in any anatomic site of the human body. The assessment of malignant potential of these tumours is still debated, as none of the proposed risk stratification systems is widely accepted. PEComas may be one of the many manifestations of Li-Fraumeni syndrome.
